# Supplementary material for: Effect of Curcumin as Feed Supplement on Immune Response and Pathological Changes of Broilers Exposed to Aflatoxin B1
Source: Biomolecules. 2022 Aug 26;12(9):1188. doi: 10.3390/biom12091188 (PMC9496629; doi:10.3390/biom12091188)
Supplement: Supplementary file 1 [file biomolecules-12-01188-s001.zip › biomolecules-1808942-supplementary.pdf]

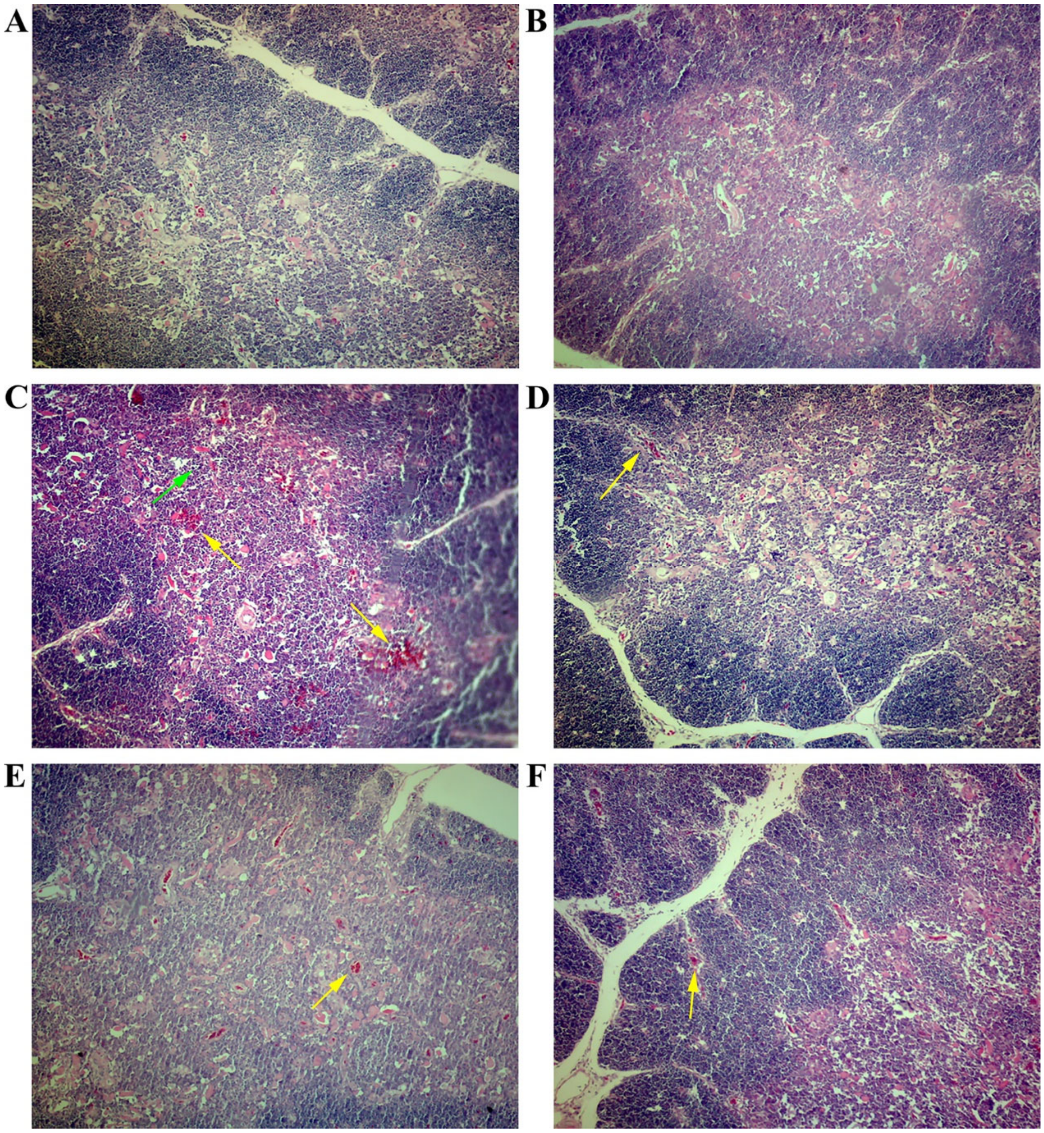

Figure S1. Histopathological observation of AA broilers thymus slices (HE, 100X) at 28 days was shown (n = 8). Yellow arrows represent tissue congestion and green arrows show nuclear fragments. (**A**) Control; (**B**) Curcumin control; (**C**) AFB1; (**D**) Curcumin I; (**E**) Curcumin II; (**F**) Curcumin III.

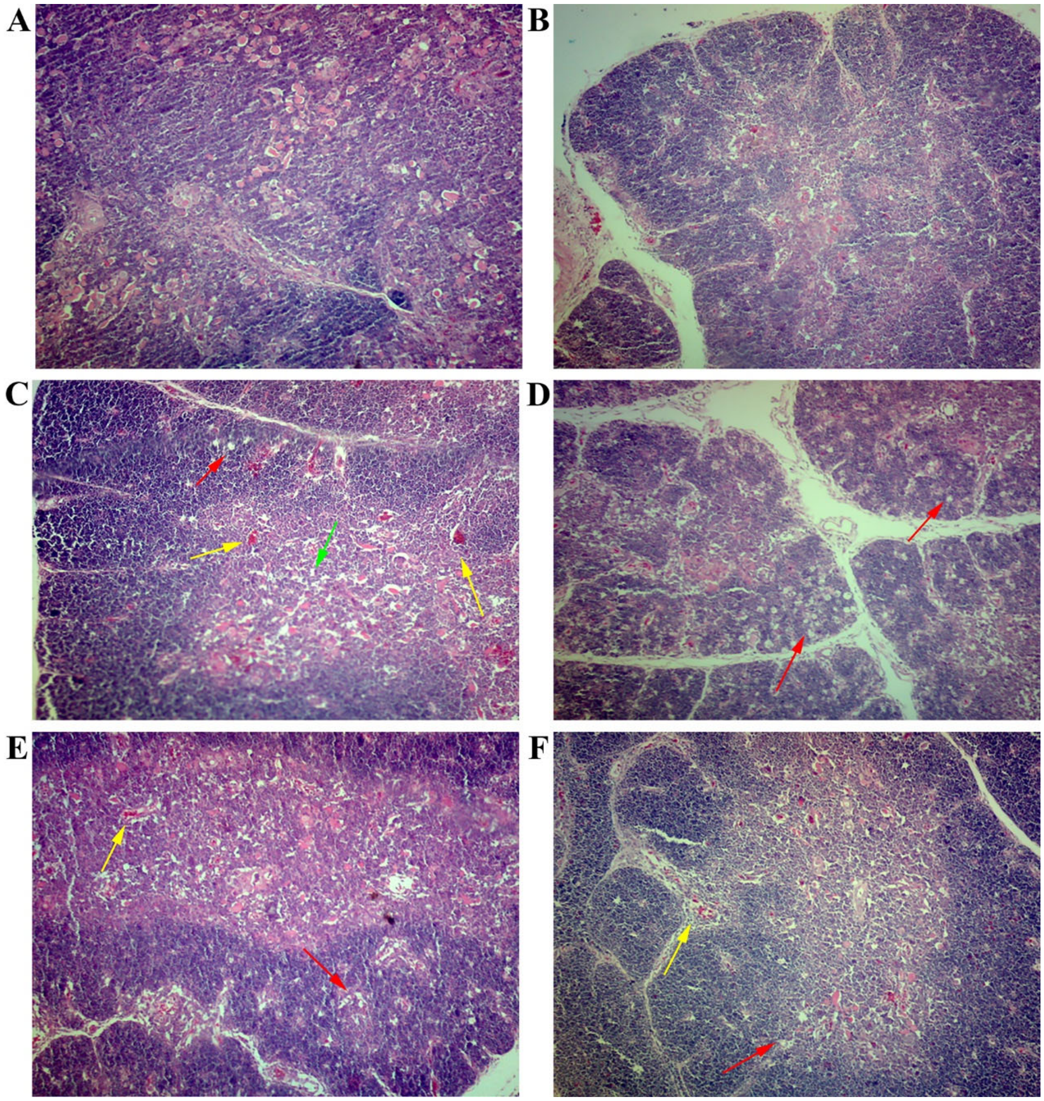

Figure S2. Histopathological observation of AA broilers thymus slices (HE, 100X) at 35 days was shown (n = 8). Yellow arrows represent tissue congestion, green arrows show nuclear fragments and red arrows represent cavity caused by the removal of necrotic tissue. (**A**) Control; (**B**) Curcumin control; (**C**) AFB1; (**D**) Curcumin I; (**E**) Curcumin II; (**F**) Curcumin III.

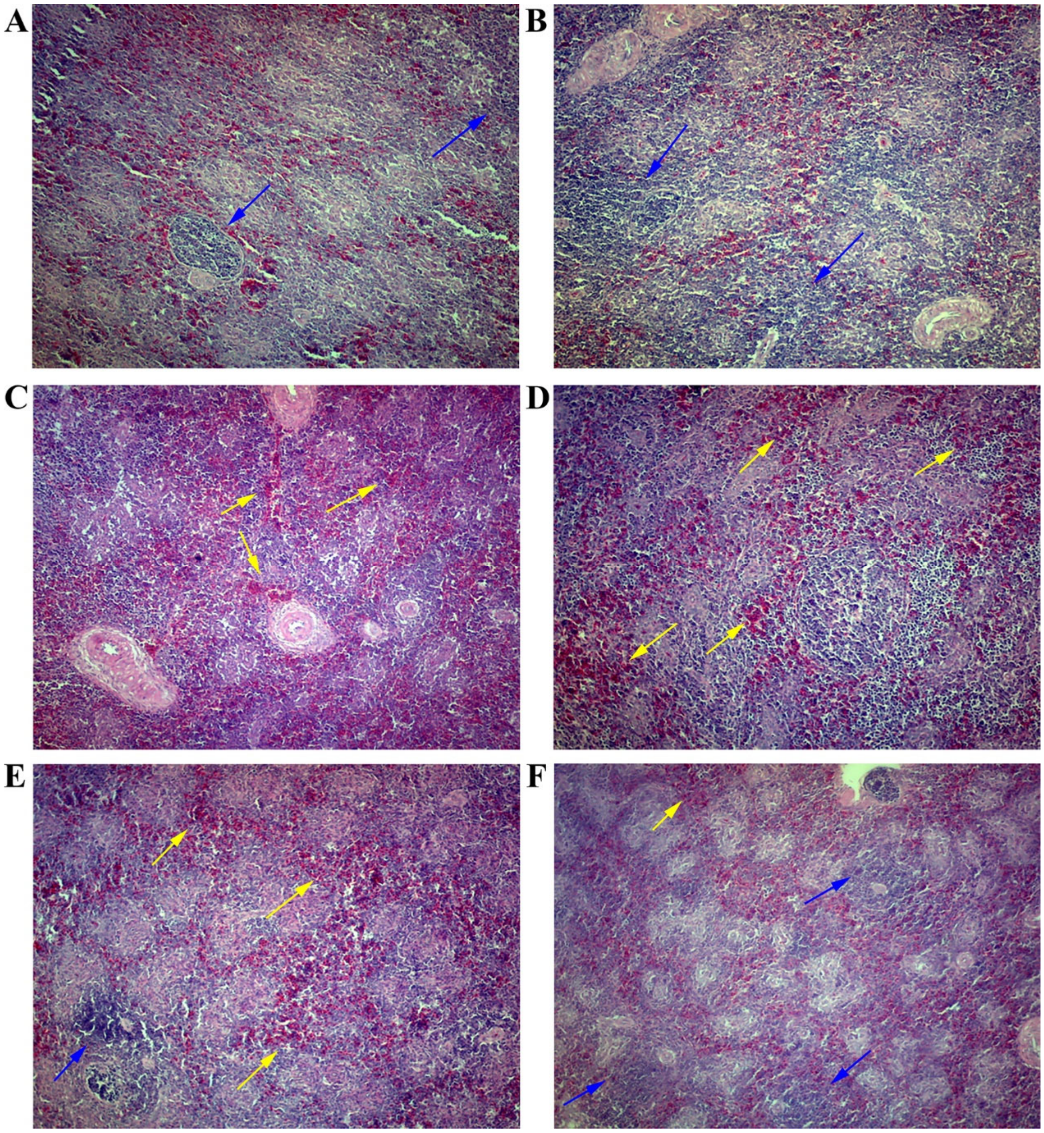

Figure S3. Histopathological observation of AA broilers spleen slices (HE, 100X) at 28 days was shown (n = 8). Yellow arrows represent tissue congestion and blue arrows lymphocytes. (**A**) Control; (**B**) Curcumin control; (**C**) AFB1; (**D**) Curcumin I; (**E**) Curcumin II; (**F**) Curcumin III.

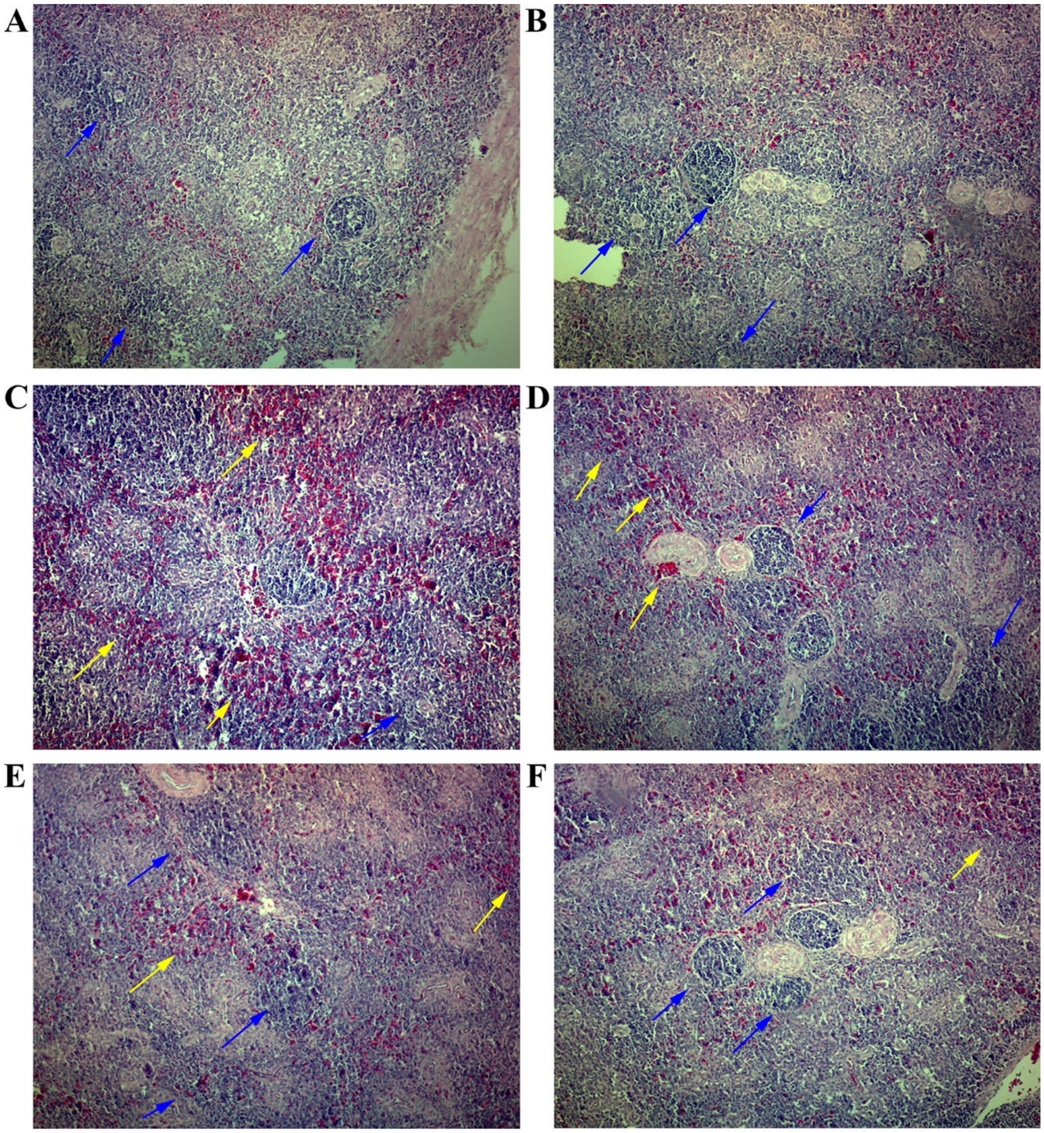

Figure S4. Histopathological observation of AA broilers spleen slices (HE, 100X) at 35 days was shown (n = 8). Yellow arrows represent tissue congestion and blue arrows lymphocytes. (**A**) Control; (**B**) Curcumin control; (**C**) AFB1; (**D**) Curcumin I; (**E**) Curcumin II; (**F**) Curcumin III.

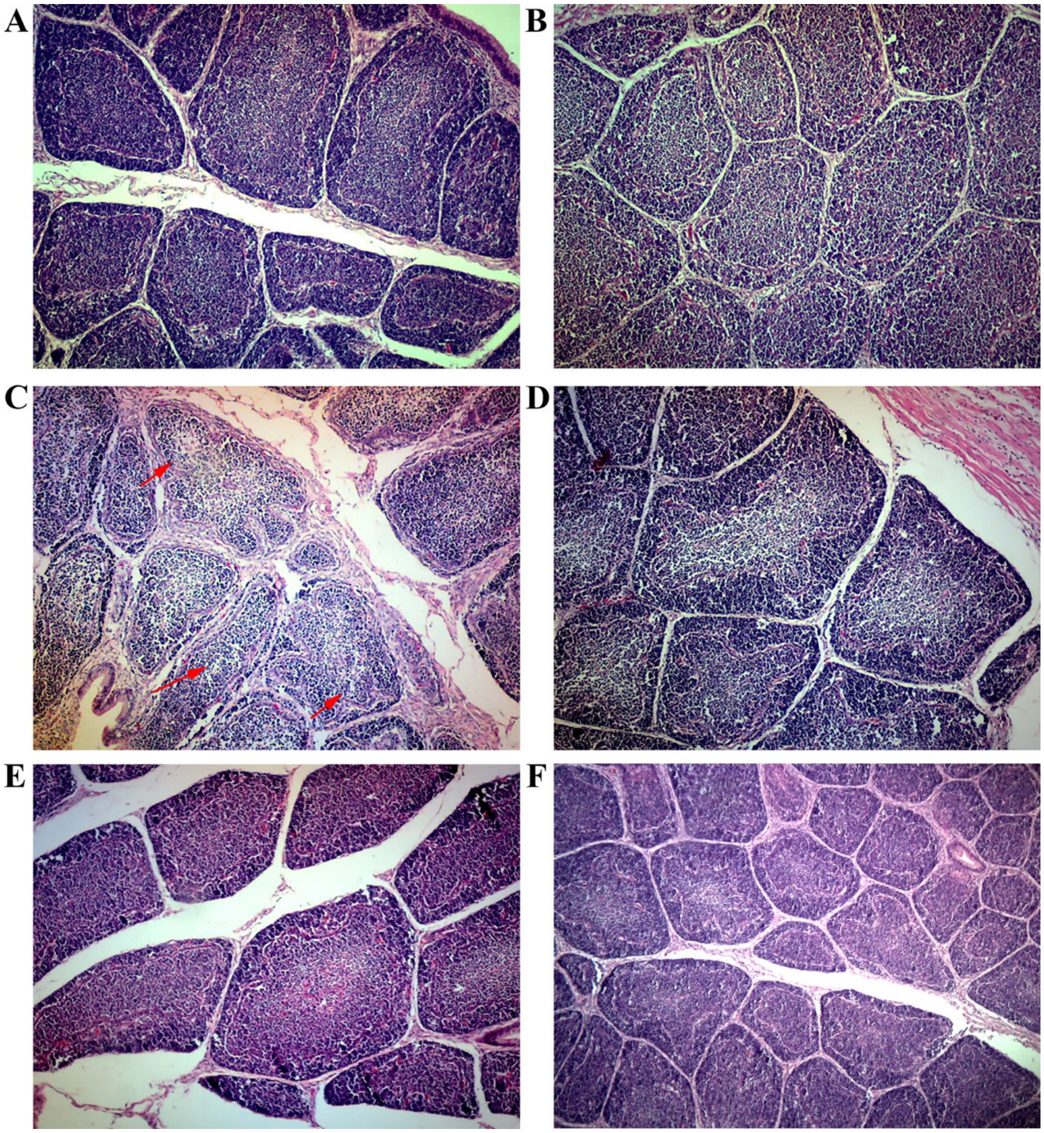

Figure S5. Histopathological observation of AA broilers bursa of Fabricius slices (HE, 100X) at 28 days was shown (n = 8). Red arrows represent sparseness and disarrangement of lymphocytes. (**A**) Control; (**B**) Curcumin control; (**C**) AFB1; (**D**) Curcumin I; (**E**) Curcumin II; (**F**) Curcumin III.

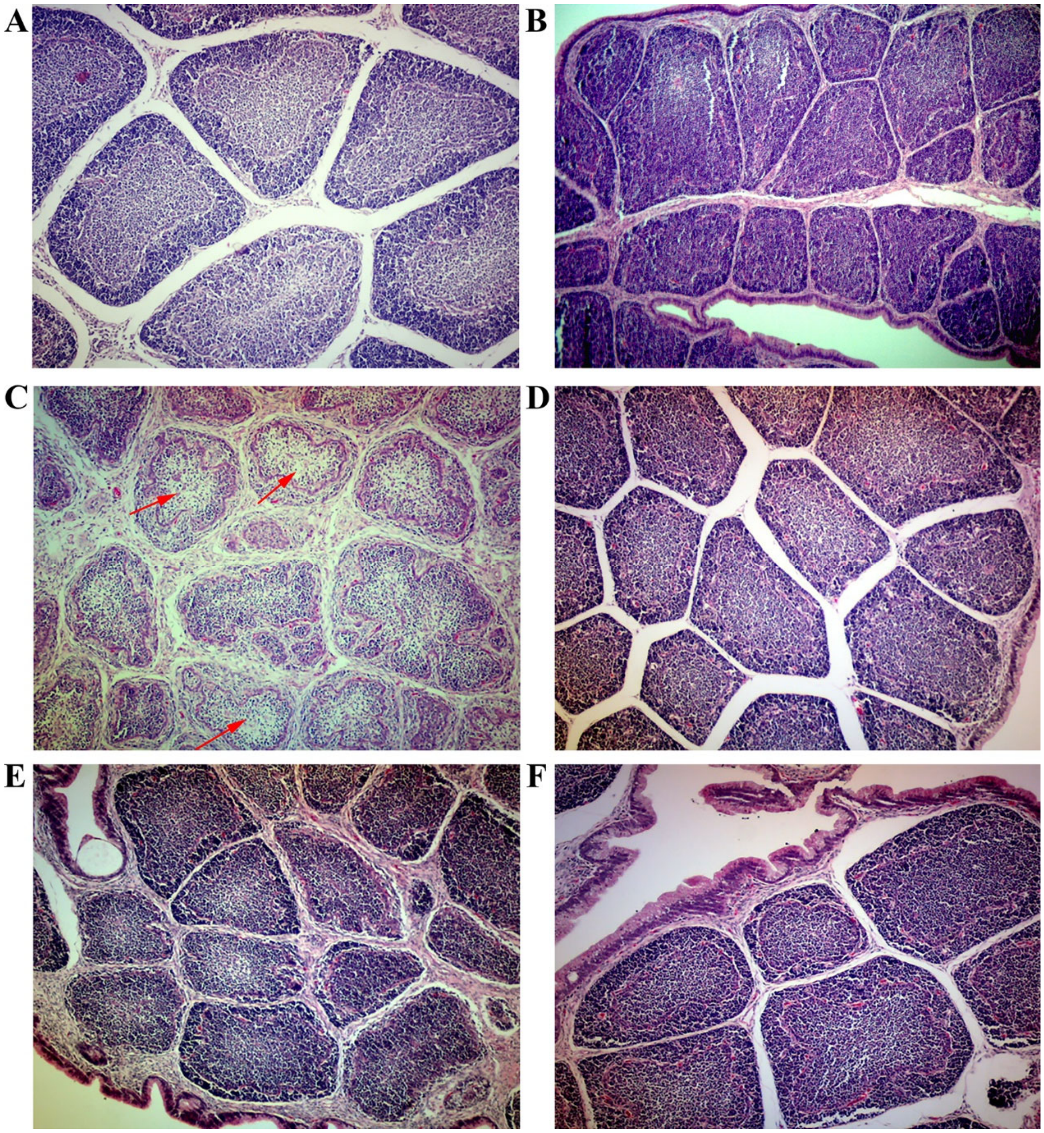

Figure S6. Histopathological observation of AA broilers bursa of Fabricius slices (HE, 100X) at 35 days was shown (n = 8). Red arrows represent sparseness and disarrangement of lymphocytes. (**A**) Control; (**B**) Curcumin control; (**C**) AFB1; (**D**) Curcumin I; (**E**) Curcumin II; (**F**) Curcumin III.
